# Right Anterior Sectionectomy for Hepatic Helminthic Infection Mimicking Cholangiocarcinoma: A Case Report

**DOI:** 10.1155/cris/9902430

**Published:** 2026-10-12

**Authors:** James Tai, Benjamin Shen, Khashayar Asadi, Marcel Leroi, Marcos Vinicius Perini

**Affiliations:** ^1^ Department of Surgery Austin Health Heidelberg Victoria Australia austin.org.au; ^2^ Faculty of Medicine Dentistry and Health Sciences The University of Melbourne Parkville Victoria Australia unimelb.edu.au; ^3^ Department of Pathology Austin Health Heidelberg Victoria Australia austin.org.au; ^4^ Department of Microbiology Austin Health Heidelberg Victoria Australia austin.org.au

**Keywords:** case report, cholangiocarcinoma, liver resection, nematode infection

## Abstract

We present the case of a patient with an unexpected pathological finding of degenerate roundworm infection on histology for a liver resection for presumed intrahepatic cholangiocarcinoma. Helminthic infection of the liver is uncommon in developed and metropolitan regions and this patient had no risk factors for such. The only potentially risk factor for complications of the infection was the presence of long‐term immunosuppression in the setting of Crohn’s disease. The imaging, including computer tomography (CT), magnetic resonance imaging (MRI), and fluorodeoxyglucose positron emission tomography (FDG‐PET) scan, was suggestive of a malignant process and thus the patient was consented and brought forward for segments five and eight liver resection. Interestingly the frozen section analysis and formal pathology confirmed a helminthic infection. The patient made an unremarkable recovery and remains well to date. Liver helminth infection mimicking malignancy is rare, however should always be considered as a differential diagnosis for atypical liver imaging findings.

## 1. Introduction

Liver helminth infection is an uncommon pathology in developed areas [1]. Given this, it is often overlooked as a diagnostic consideration for atypical liver lesions on imaging particularly with the prevalence and significance of primary biliary malignancy. These parasitic infections are also a well‐established association with biliary tree malignancy, predominantly in Asia and other parts of the developing world [2–5]. Nematodes (roundworms) are classified within a broader group of helminths, which also includes cestodes (tapeworms) and trematodes (flukes) that infest the human gastrointestinal system [6]. The three most prevalent pathological hepatic Helminth infections linked to cholangiocarcinoma (CCA) are *Opisthorchis viverrini*, *Clonorchis sinesis*, and *Schistosomiasis japonica* [7, 8]. Conversely, in developed countries, the main pathological associations with CCA are primary sclerosing cholangitis, diabetes, and choledochal cyst [9, 10].

Faecal microscopy for the presence of eggs is a validated and readily available method for assessment of the presence of viable helminths [6]. Faecal polymerase chain reaction (PCR) analysis for helminthic genetic material in stool is also useful and typically more sensitive [11]. Liver parasite antibodies can also be detected in serum, saliva, and urine although the availability of commercial assays is very limited. Furthermore, active and past infection are challenging to distinguish based on this alone [12].

The gold‐standard radiological assessment for a presumed CCA involves multimodal imaging [10]. The Liver Cancer Study Group of Japan published clinical practice guidelines for intrahepatic CCA in 2022 [13]. In this guideline, they gave strong recommendation for abdominal ultrasound (US), computer tomography (CT), and magnetic resonance imaging (MRI) as effective imaging modalities for the diagnosis of intrahepatic CCA[13]. Fluorodeoxyglucose positron emission tomography (FDG‐PET) scanning for intrahepatic CCA larger than 1 cm has a sensitivity reported as high as 100% [14] and a specificity of 90% [15]. The Japanese clinical practice guideline recognised FDG‐PET as effective in differentiating benign and malignant processes although was not considered gold standard given resource and availability [13].

There were several learning points to be taken from this case, including that in a patient with longstanding immunosuppression, all potential atypical aetiologies, particularly infective, need to be considered. Furthermore, although multiple imaging modalities, each with high sensitivity and specificity, were suggestive of an intrahepatic CCA, a liver helminth infection can give similar radiological appearances. Whilst liver helminthic infection is rare in the developed world, it is still required to be considered as a differential diagnosis, even in cases where patients have no history of concerning travel or exposure.

## 2. Case Presentation

A female patient aged in her fifties was referred to our tertiary institution with a segment eight liver lesion identified on magnetic resonance enterography (MRE) as part of their routine surveillance for Crohn’s disease. Her medical history was significant for Crohn’s disease, for which she was on fortnightly Infliximab with quiescent disease clinically, radiologically and at endoscopy, as well as asthma controlled with combination inhaled therapy and Paget’s disease on anti‐resorptive therapy. The patient had undergone previous laparoscopic cholecystectomy, laparoscopic appendicectomy, and open ileocolic resection for Crohn’s disease.

The patient lived in a semi‐regional town where she was raised and could not recall any significant exposure to farming or livestock. She currently lived with her family and a domestic cat and dog. The patient worked in a government support agency having previous employment at a supermarket and childcare. She was a reformed smoker, consumed minimal alcohol, and was functionally independent. There were no high‐risk travel or other practices for transmissible infection.

At the time of referral, the patient was clinically well and the liver lesion was an incidental finding. There was no history of abdominal pain, constitutional symptoms, change in bowel habit, jaundice, or steatorrhoea. The most recent relevant pathology included a haemoglobin of 141 g/L (range 115–155 g/L), platelet count 277 × 10^9^/L (range 150–400 × 10^9^/L), eosinophil count 0.2 × 10^9^/L (range 0.0–0.5 × 10^9^/L), creatinine 60 μmol/L (range 45–90 μmol/L), bilirubin 14 μmol/L (range 1–20 μmol/L), ALT 33 U/L (range 5–35 U/L), CEA 4.2 μg/L (range 0–5 μg/L), alpha fetoprotein 2.3 kU/L (range 0–7.4 kU/L), Ca 125 28 U/mL (range 0–35U/mL), Ca 19.9 10 U/mL (range 0–35 U/mL), and INR 0.9. A colonoscopy performed in the month preceding referral as part of surveillance for Crohn’s disease was essentially normal with unremarkable biopsy results. A multiphase CT was arranged for further evaluation (Figure 1), which demonstrated a 21 mm hypodense, non‐enhancing lesion in segment eight suspicious for a primary intrahepatic CCA with no abdominal lymphadenopathy or evidence of metastatic disease. Following discussion in a multidisciplinary meeting, the decision was made for further imaging with MRI with Primovist enhancement (Figure 2) and an FDG‐PET scan (Figure 3). The MRI revealed a 19 mm^2^ × 13 mm^2^ segment eight lesion that was T2 hyperintense, demonstrated diffusion restriction, and no uptake of Primovist, highly suggestive of CCA. There was evidence of localised metabolic activity in the segment eight liver lesion on the FDG‐PET, further supporting the malignant differential, and as such, it was deemed to be locally resectable with sacrifice of the right anterior Glissonian pedicle.

**Figure 1:**
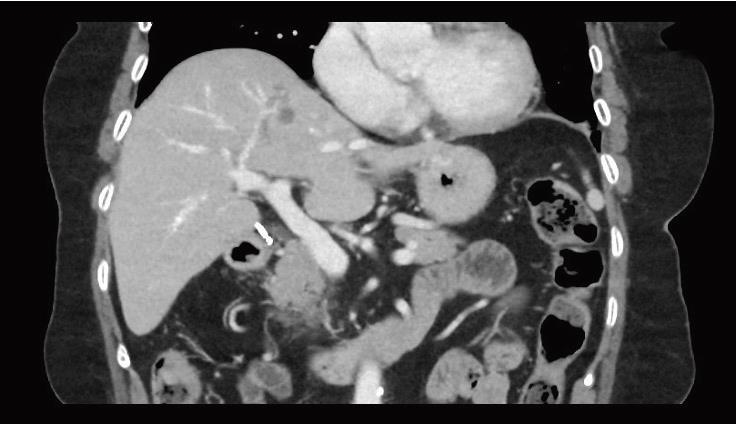
Coronal slice of upper abdominal CT in the portal venous phase demonstrating a 18 mm^3^ × 12 mm^3^ × 21 mm^3^ irregular hypoenhancing lesion in segment eight.

**Figure 2:**
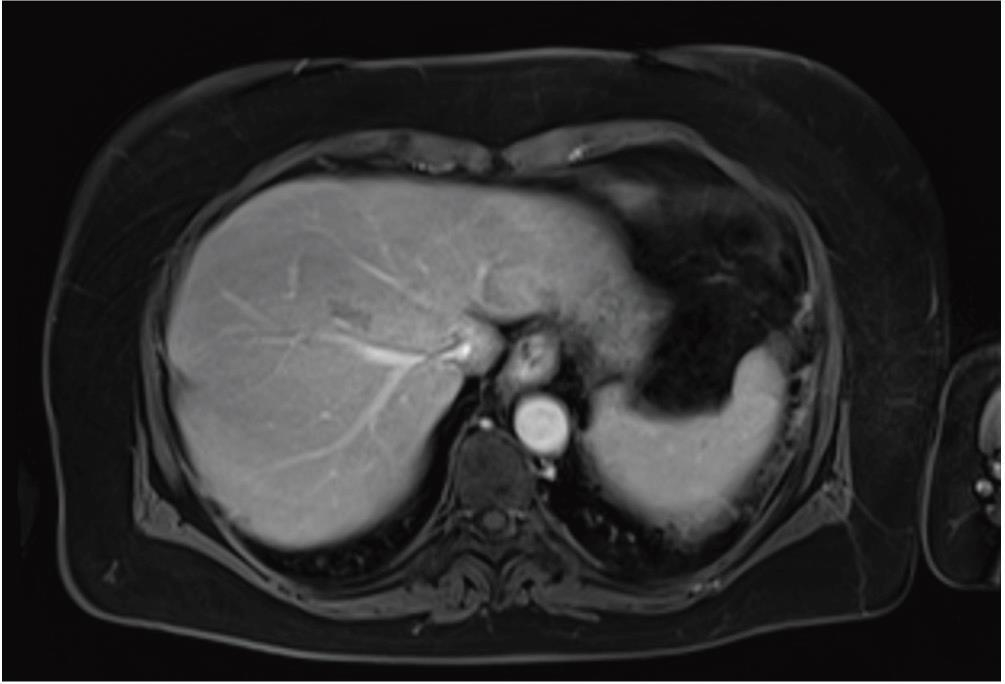
Axial slice of T1‐weighted MRI of the liver with a hypointense segment eight lesion measuring 19 mm^2^ × 13 mm^2^. The lesion further demonstrated T2 hyperintensity, diffusion restriction and no uptake of Primovist contrast.

**Figure 3:**
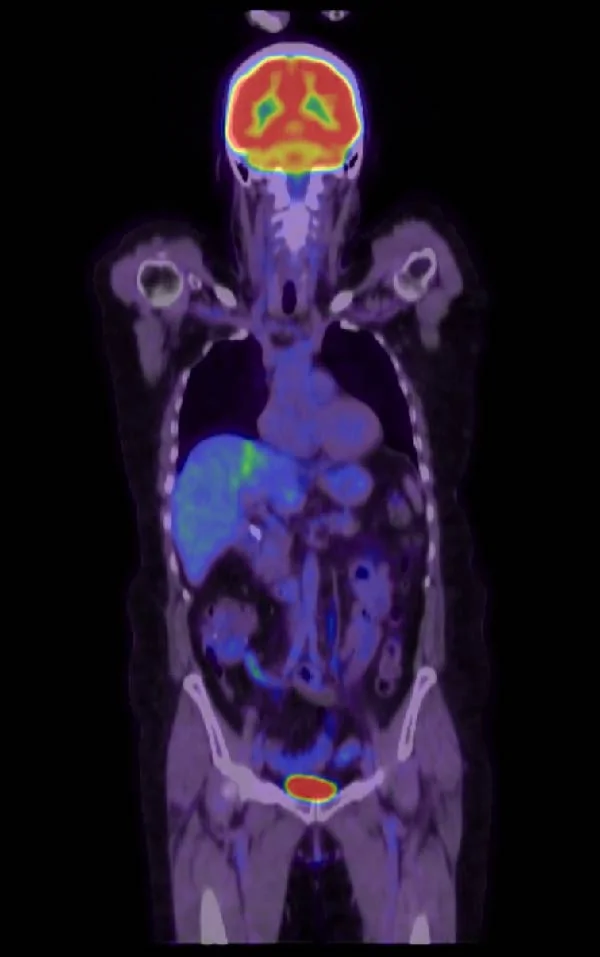
Coronal slice of FDG‐PET following IV administration of 264 MBq F‐18 FDG and a 71 min uptake time demonstrating moderate FDG avidity in segment eight of the liver with no other FDG‐avid lesion identified.

With the latest investigations, the patient was discussed once more in a multidisciplinary meeting with the consensus to proceed with open resection given the consistent imaging findings suspicious for a locally resectable intrahepatic CCA. Percutaneous biopsy was considered; however, given the extended time course between referral, further investigations, and repeat multidisciplinary discussion, an upfront resection was favoured. The treatment intent was deemed curative with the surgical plan for segments five and eight resection with potential formal right hepatectomy if required. Formal liver volumetrics were performed, and it was deemed the patient would have adequate functional liver remnant to proceed with both surgical options.

The patient underwent an uncomplicated open resection of liver segments five and eight using a right anterior Glissonian approach (Figure 4). Intraoperatively, an inflammatory lesion was identified at the base of the right anterior sheath. Frozen section analysis of the lesion confirmed the absence of malignancy, revealing granulomatous inflammation with clear surgical margins. The patient proceeded to an uncomplicated in hospital recovery and was discharged home on post‐operative day six.

**Figure 4:**
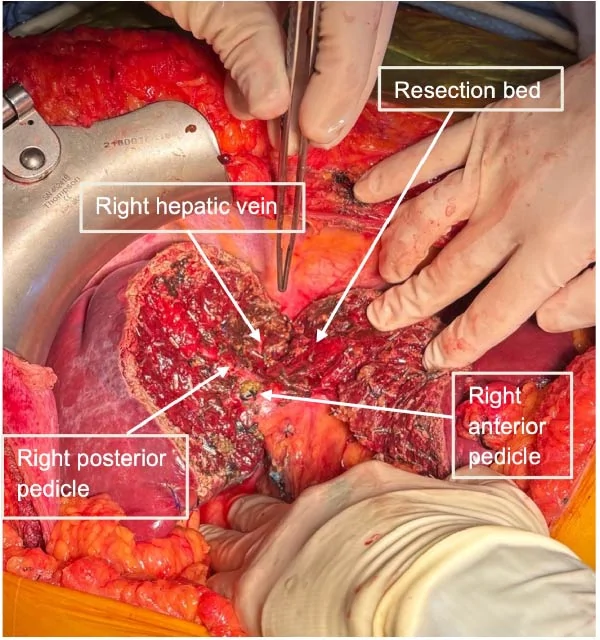
Intraoperative image post completion of resection demonstrating the extent of resection and key associated anatomical structures.

Final histology from the liver resection confirmed mass‐forming necrotising granulomas with Charcot‐Leyden crystals and ghosted outlines consistent with a non‐viable helminth (Figures 5 and 6). Fungal and mycobacterial stains were negative, and the background liver was non‐cirrhotic.

**Figure 5:**
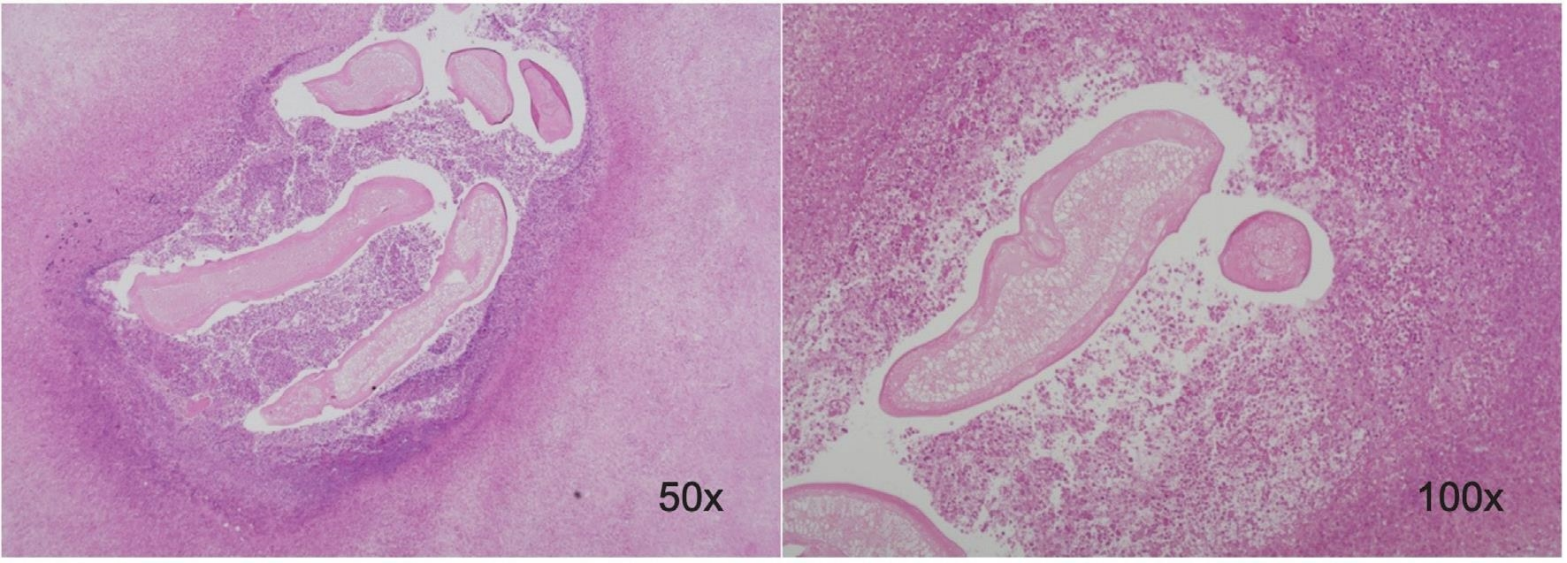
Haematoxylin & Eosin (H&E) stains at 50x and 100x magnification showing focal cavitation with necrotic material and ghosted outlines of Helminths.

**Figure 6:**
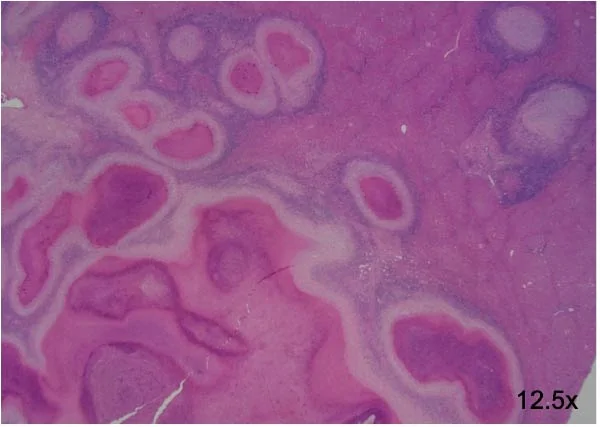
H&E stain at 12.5x magnification showing coalescing necrotising granulomas with palisading histiocytes and surrounding mixed inflammatory cells.

The serology and faecal assessment for helminth infection were performed when the final histopathology result was available. Microscopy for ova, cysts, and parasites revealed no detectable ova or eggs. An attempt to molecularly identify the parasite by sequencing extracted DNA from the paraffin block was unsuccessful.

The patient was administered a single dose of albendazole as an inpatient following assessment by the Infectious disease specialists. A further dose of albendazole was prescribed at 4 weeks post‐operatively; however, no other dedicated therapy has been instituted since the hepatic resection.

The patient was seen in the outpatient clinic at 2 and 4 weeks following the resection without any major concerns. They had no significant abdominal pain, return of normal bowel habit and no symptoms suggestive of a post‐operative infection. The surgical wound and drain sites were well‐healed and repeat pathology performed on day 14 post‐operatively showed a normal white cell count and down trending C‐reactive protein (CRP). Infliximab was withheld as per institutional protocol following major abdominal surgery. The patient has ongoing follow‐up with both Infectious disease specialists and the Hepatobiliary surgery team.

## 3. Discussion

Whilst the carcinogenic association between intrahepatic parasitic infection and CCA is well established, this is one of few reported cases for dormant helminthic infection rather mimicking a primary intrahepatic malignancy [2, 4]. More commonly, helminthic infections present as hepatic abscesses, stone formation, or periductal fibrosing disease predisposing to cholangitis [1, 11].

A case study published out of Japan of *Fascioliasis* presenting as a liver mass has previously been reported [16]. In that case, the patient underwent a CT as the only dedicated imaging modality. Similarly to the case reported here, the patient did not have significant risk factors for helminthic infection, and both active adult parasites and eggs were not detected in faecal or liver specimens. Roundworm infection leading to liver abscess formation has also been well‐published. In one case series, at‐risk population patients were managed with combinations of medication, endoscopy, and surgery, and there was evidence of both active and inactive helminth infection on pathology [17].

Hepatic helminthic infection of the liver may induce inflammatory change via a multitude of mechanisms including tissue damage from the migration of larvae, parasites causing biliary obstruction, as well as via the host immunological response [6, 18]. Interestingly, some trematodes can inhabit the biliary tree for close to 20 years, although, as with the case presented here, the majority of patients with roundworm infection are asymptomatic [6]. In 10%–20% of cases, patients with active helminthic infection will have an elevated serum bilirubin [6]. Eosinophilia is also associated with the larval stages of infection as the organism is migrating extraluminally [6], although in the case presented, both bilirubin and eosinophil count were within normal limits. This is likely as the infection had resolved by the time of testing. With regards to the presented case, with a high index of suspicion for helminthic hepatic infection, faecal assessment for adult worms, eggs, or larvae could also have been conducted pre‐operatively to determine if there was viable intestinal infestation at the time, although notably these were not detected in a faecal testing performed following resection.

The recommended treatment for suspected helminth infection is anti‐helminthic therapy, such as albendazole, as was prescribed in this case, with up to 97% efficacy reported [19].

Achieving a diagnosis of inactive helminth infection in this case would have been very challenging pre‐operatively. Given that the working diagnosis was for an intrahepatic CCA based on comprehensive radiological assessment, as well as the paucity of clinical and biochemical evidence of helminthic infection, the resection was undertaken to treat what was believed to be the most likely, and most serious, pathology.

## Funding

No funding was received for this study. Open access publishing was facilitated by The University of Melbourne through the Wiley — The University of Melbourne agreement with the Council of Australasian University Librarians.

## Ethics Statement

Formal ethics approval was not required for this study. This single‐patient case report is a retrospective description of routine clinical care and does not constitute human research as defined by the National Health and Medical Research Council (NHMRC) *National Statement on Ethical Conduct in Human Research (2023)*. Consistent with the Austin Health institutional policy, single case reports do not require review by the Austin Health Human Research Ethics Committee (HREC), provided written informed consent for publication is obtained from the patient. Written informed consent for publication of this case report and any accompanying images was obtained from the patient, and all data have been de‐identified. The authors are accountable for all aspects of the work in ensuring that questions related to the accuracy and integrity of any part of the work are appropriately investigated and resolved.

## Consent

The patient provided written informed consent for publication of this case report and any accompanying images.

## Conflicts of Interest

The authors declare no conflicts of interest.

## Data Availability

The data that support the findings of this study are available from the corresponding author upon reasonable request.
